# Unlocking the WHO’s Age-Friendly Healthcare Principles: Portugal’s Quest and Recommendations

**DOI:** 10.3390/ijerph20227039

**Published:** 2023-11-09

**Authors:** Jéssica Tavares, Gonçalo Santinha, Nelson Pacheco Rocha

**Affiliations:** 1 GOVCOPP, Department of Social, Political and Territorial Sciences, University of Aveiro, 3810-193 Aveiro, Portugal; g.santinha@ua.pt; 2 IEETA, Department of Medical Sciences, University of Aveiro, 3810-193 Aveiro, Portugal

**Keywords:** ageing, age friendly, healthcare, health policy, Portuguese National Health Service

## Abstract

Countries worldwide are grappling with a pressing demographic challenge characterized by a growing older population. This poses a significant healthcare dilemma, presenting challenges for healthcare systems and providers. To address these challenges, the World Health Organization (WHO) has devised a set of Age-Friendly Principles, aimed at optimizing healthcare provision for older people. This article delves into the current state of healthcare adaptation for older adults in Portugal and assesses the implementation of the WHO Principles. Case studies were conducted in three distinct regions of Portugal, involving semistructured interviews with key decision makers from both the healthcare sector and organizations wielding direct influence over health policies (*n* = 11). A comprehensive content analysis was conducted employing the webQDA software. The findings unveiled a noteworthy trend in which most interviewees displayed limited familiarity with the WHO Principles. Nevertheless, all interviewees acknowledged the need to adapt the healthcare system accordingly. Strengths were identified, primarily within the healthcare management system, but noteworthy gaps were also revealed, particularly in terms of facility preparedness and professional training. Interviewees proposed various interventions to enhance age-friendly healthcare provision; however, they concurrently pinpointed challenges related to human resources, infrastructure, and financial management. In their concluding recommendations, interviewees underscored the development of tools to facilitate the application and evaluation of the WHO Principles, as well as the development by the WHO of an accreditation system to encourage the application of the principles in healthcare providers across the world.

## 1. Introduction

Demographic trends comprise a set of challenges for healthcare systems and policymakers. These challenges encompass not only an increase in public health expenditure but also structural considerations concerning the nature of care provision and the configuration of healthcare services [1].

As people across regions are experiencing longer lifespans, the prevalence of noncommunicable diseases and disabilities is increasing, especially in aging populations. Notable among these chronic conditions are ischemic cardiovascular disease, neurodegenerative diseases, chronic obstructive pulmonary disease, diabetes, cancer, and depression [2]. The onset of the COVID-19 pandemic has worsened the vulnerability of older adults [3], exposing a heightened susceptibility to SARS-CoV-2 infection due to physiological changes that come with aging and an increased likelihood of severe disease outcomes among older adults with comorbidities [4]. 

This trend has led to greater use of healthcare services [5] and longer hospital stays [6], which, in turn, have increased the per capita health expenditure levels, aggravating the financial sustainability of healthcare systems [7]. In Portugal, the percentage of individuals aged over 65 increased from 18.7% to 22.4% between 2011 and 2021 [8]. This change has had a significant impact on current health expenditure, rising from EUR 1623.05 per capita to EUR 2049.89 per capita between 2011 and 2020, equivalent to a 0.83% increase in GDP [9]. Similarly, other European countries, such as Italy, Greece, and Spain are also experiencing demographic aging and a gradual increase in current health expenditure, with the exception of Ireland, which saw a 3.5% decrease in GDP from 2011 to 2021. With a growing older population, governments are grappling with increased demand for public health services and economic resource constraints, jeopardizing the sustainability of the healthcare system [5].

As individuals age and their health conditions deteriorate, the increase in demand for healthcare services and healthcare expenditure leads healthcare organizations to tailor their approach to cater to the needs of older adults [10]. To enhance the autonomy and quality of life of older adults, it is crucial to consider measures of positive discrimination in healthcare. In 2004, the World Health Organization (WHO) defined the Age-Friendly Principles within the realm of primary healthcare (PHC) [10]. Subsequently, a toolkit was developed to facilitate the implementation of these principles, with the aim of extending their application to hospital healthcare (HHC) [11]. These proposed principles encompass the following aspects [10]:

1. Information, education, communication, and training, which includes staff training in clinical management and approaches to patient and family education;
2. Healthcare management systems, i.e., adapting procedures to address the unique needs of older adults and ensuring continuity of care through updated medical records available at each visit;
3. Physical environment, i.e., clean and comfortable healthcare centers that apply the principles of universal design to the greatest extent possible.

However, a systematic review [12] aiming at understanding the global implementation of the WHO Principles in healthcare revealed that their adoption is not as widespread as desired, indicating limited use of the WHO toolkit. Notably, international studies evaluating the application of the WHO Principles predominantly focus on primary care [13,14] or hospital care [15], without conducting a comprehensive assessment of the National Health Service (NHS). Moreover, within the European context, studies on this subject are scarce, often deviating from the precise utilization of the WHO Principles and failing to assess their implementation in healthcare [16,17,18,19].

In Portugal, the median age already ranks among the highest within the Organization for Economic Cooperation and Development (OECD) member countries [20]. Given this demographic context, the WHO Principles have been integrated into the National Strategy for Active and Healthy Aging 2017–2025 (NSAHA) [21], a strategic initiative developed by the Directorate-General for Health. The primary objective is to stimulate the adaptation of healthcare services, thereby enhancing the quality of care, fostering participation, promoting independence, and preserving the dignity of older adults [21]. To this end, a collaborative effort involving the Ministry of Health, various administrative entities, municipalities, and the social security system has been devised to expand the WHO initiative as part of the management of comorbidity processes [21].

A document analysis conducted in Portugal has revealed that health guidance documents address older adults to a lesser extent than expected, with the WHO Principles being addressed in a single document, namely the NSAHA [22]. Additionally, a quantitative study conducted in Portugal indicated that the implementation of the WHO Principles within the NHS fell short of expectations [23]. However, the precise guiding model for effectively applying these principles within the NHS, along with the requisite conditions and resources, remained unclear. This study seeks to comprehensively understand the current state of healthcare adaptation in Portugal for older adults, while also highlighting necessary improvements and the essential resources needed to establish age-friendly healthcare within the Portuguese NHS.

## 2. Materials and Methods

### 2.1. Research Questions

In order to gain insight into the initiatives within the NHS related to healthcare for older adults, as well as the implementation of the Age-Friendly Principles and the necessary mechanisms for their operationalization, the following research questions were formulated:

RQ.1. How are the Age-Friendly Principles perceived by local and regional decision makers?

RQ.2. What is the current approach of the NHS toward age-friendly healthcare services?

RQ.3. What initiatives are presently being employed within the NHS to provide age-friendly healthcare services?

RQ.4. How can the Age-Friendly Principles be effectively implemented within the NHS?

RQ.5. Which resources and measures are required for the successful operationalization of the Age-Friendly Principles in the NHS?

To address these research questions, case studies were conducted, involving interviews with key stakeholders at the local and regional levels.

### 2.2. Case Studies

The analysis was carried out across three distinct case studies. Recognizing the highly diverse territorial landscape, efforts were made to broaden the understanding of geographical contexts that presented different structural conditions for development and, therefore, had the potential to generate additional insights for the ongoing research. The study focused on three NUTS 2 Regions (Nomenclature of Territorial Units for Statistics), encompassing two subregions located along the coastal areas and one situated in the interior of Portugal. These diverse geographical settings include medium-sized cities and share areas of influence with similar healthcare providers, yet they exhibit contrasting demographic and economic profiles.

The first case study is part of the Global Network of Age-Friendly Cities, where actions pertaining to health promotion and the adaptation of healthcare services for older adults are anticipated. In the second case study, a substantial number of projects have been developed in the realm of age-friendly environments. Lastly, the third case study boasts one of the highest aging indices at the national level and is unique in its inclusion of a local health unit (LHU). Local health units integrate different levels of care (health centers and hospitals) into a single entity with unified management, facilitating the improved development of responses and the planning of local and regional infrastructures. This integration also enhances their coordination with existing social network facilities within the respective territories.

### 2.3. Participants

This research sought to engage decision makers within the healthcare sector who wield direct influence over the implementation of health policies. Understanding the perspectives and experiences of these stakeholders provides a concrete view not only of the guiding model for healthcare providers’ adoption of the Age-Friendly Principles but also of the governance mechanisms held by key institutions in the healthcare sector and the limitations that shape their actions. To this end, key stakeholders at both the regional and local levels were consulted within the three case studies. This encompassed representatives from regional health administrations (RHA), primary healthcare center groups (PHCG), HHC, and LHU.

Nevertheless, at the local level, there are other significant actors in the healthcare field whose perspectives are essential to analyze. These actors are known to play an active role in, or at least influence, decision-making processes within the healthcare domain [24,25]. In this context, Decree-Law No. 23/2019, January 30, has conferred new competences upon local governments in the field of PHC, including responsibilities related to the maintenance, conservation, and equipping of healthcare-unit facilities. Consequently, representatives from intermunicipal communities (voluntary associations of municipalities) in the three case studies, bearing healthcare responsibilities, were also consulted during the research process.

The process of recruiting participants for the three case studies was executed through institutional emails. Each entity involved in the case studies received an individual email containing a concise explanation of the study, its objectives, and the required ethics committee authorization. A response time of one month was stipulated, after which telephone contacts were initiated with the entities.

Upon receiving authorization, collaboration requests were conveyed via email, accompanied by a brief message introducing the study and outlining the objectives of the interview. During this correspondence, potential interview dates were proposed, offering both in-person and online formats to accommodate the preferences of the interviewees. Subsequent biweekly follow ups were conducted through phone calls and emails to reinforce the interview scheduling request.

### 2.4. Data Collection

The data-collection method involved semistructured individual interviews. This approach enables the researcher to direct the conversation towards predefined objectives while still granting interviewees a comfortable degree of freedom to discuss aspects of personal interest. While this interview type requires prior script preparation to serve as a guide, it also allows flexibility in changing the question sequence and incorporating or omitting questions that may become relevant during the interview. The interviews were conducted following a bottom-up approach. The intention was to gradually introduce interviewees to the topic, establishing a conversational foundation that allowed for a deeper understanding of their perspectives on the subject. The interview script encompassed descriptive and explanatory questions, organized into four blocks: Block (A) Interview justification and ethical considerations; Block (B) Development; Block (C) Final questions; and Block (D) Acknowledgment and Validation.

Block A started with a brief introduction by both the interviewer and interviewee, followed by a concise explanation of the interview’s objectives. The importance of maintaining the anonymity and confidentiality of the interview content was emphasized, and permission was sought to record the interview for analysis.

Block B was dedicated to exploring the concept under study. It delved into the primary challenges faced when providing care to older adults and evaluated the current capacity of healthcare providers to meet their needs. Furthermore, it discussed the adaptation of healthcare providers to the WHO Age-Friendly Principles [10]. This Block consisted of a series of key and guiding points, aligned with a set of objectives. The first one, of a general and introductory nature, aimed to gauge the interviewees’ understanding of the age-friendly healthcare concept and the WHO Principles. The second one aimed to identify person-centered initiatives implemented by healthcare providers and the main challenges encountered. The third objective assessed the current status of healthcare providers in terms of the implementation of the WHO Principles. Finally, the last and concluding objective aimed to determine how the WHO Principles could be effectively implemented and the necessary means for their operationalization within the NHS.

Block C presented a summary question to recap the topics covered during the interview and highlight key points. Additionally, interviewees were encouraged to discuss other subjects and pose any additional questions to the interviewer.

In Block D, the interviewee’s cooperation was acknowledged, and they were informed about the requirement for interview transcription for validation (postinterview).

The interviews were primarily conducted in person between January 2022 and November 2023, taking into consideration the availability of interviewees during the postpandemic recovery period and any unforeseen professional or personal circumstances. These interviews were recorded in audio format and generally lasted approximately one hour on average.

### 2.5. Data Analysis

This task involved several steps. Initially, all the gathered material, totaling eleven protocols, was transcribed by the authors. These protocols were then presented to the respective interviewees for verification, ensuring the accuracy of their statements.

Subsequently, a comprehensive analysis was undertaken to organize and categorize the data in a manner that addressed the research questions and aligned with the proposed objectives. To process the acquired data, a content-analysis technique was employed, following the four defined steps outlined by Bardin [26]: (i) preanalysis, (ii) data exploration, processing, and analysis, (iii) results’ inference, and (iv) interpretation.

The preanalysis of the interviews started with the organization and categorization of the collected data by the authors. Registration units were thus grouped into categories that were predefined based on the five research questions and into subcategories that were defined subsequently by identifying the themes present in the interviewees’ statements. The analysis grid encompasses five primary categories and ten subcategories (see Table 1).

The collected material was analyzed after establishing the classification rules. Initially, the validation and coding of all materials took place, followed by both quantitative (frequency analysis) and qualitative analysis (analysis of meanings). The analysis involved the identification of prevalent themes within the statements, isolating them to condense information and facilitate comparison and interpretation. This approach offers an overview of how different individuals approach the topic, highlighting distinctions and similarities among them [27].

The coding of materials followed the criteria of objectivity and systematicity. In other words, categories were applied consistently and unambiguously to all collected materials. To achieve this, the objectives for each category were predefined in the analysis grid. Simultaneously, the validity of the coding process was upheld by maintaining clarity and rigor within the categories to minimize ambiguity during classification. To ensure data reliability, the analysis underwent verification by all the authors.

To present the results, the data was organized with consideration for the WHO Principles, where applicable, along with a series of themes raised by the interviewees. These themes were not subject to a specific classification rule; rather, they were determined based solely on the similarity of the interviewees’ statements within each subcategory and to facilitate data representation.

The content analysis was conducted in a horizontal manner (i.e., comparing each category across various responses), quantifying the occurrence of specific themes in the interviewees’ statements and the associated meanings attributed to them. Particularly within the familiarity and initiatives categories, the count was focused on the number of interviewees who acknowledged the presence or absence of certain themes, as opposed to tallying mentions per theme. Content analysis was performed using the webQDA software [28], which aided in material organization and data coding.

### 2.6. Validity and Rigour

This study used a qualitative design to capture the current state of healthcare adaptation for older adults in Portugal and assess the implementation of the WHO Principles, with data gathered via semistructured individual interviews. Consolidated criteria for reporting qualitative research (COREQ) informed all steps in the data collection, analysis, and reporting [29]. The rigor of this study was ensured using the four criteria described by Lincoln and Guba [30]: credibility, transferability, confirmability, and dependability. Credibility was established through member checking during the interview and analysis triangulation, in which the three authors independently analyzed the transcription and reached a consensus on the results. Transferability was guaranteed by the detailed description of the findings and the relationship of the results with what was found in similar studies. Dependability and confirmability were ensured through the analysis process, where the findings were discussed, consensus was reached, and a full audit trail was maintained. Additionally, coding systems were used during the analysis process to improve dependability.

### 2.7. Ethical Considerations

The study was conducted in accordance with the Declaration of Helsinki of 1975 and the Portuguese rules of the General Law for the Protection of Personal Data, 8 August 2019. The protocol was approved by the Ethics Committee of the Regional Health Administrations (2150/CES/2021) and, according to this protocol, all subjects gave their informed consent for inclusion before they participated in the study.

## 3. Results

In total, eleven interviews were conducted with representatives of healthcare entities (*n* = 2 from RHA, *n* = 3 from PHCG, *n* = 2 from HHC, and *n* =1 from LHU) and local government (*n* = 3), all of whom were included in the three case studies. Of the total interviewees, six were female and five were male. In terms of age, nine interviewees were over fifty years old, while only two were between forty and fifty years old, the latter group having less experience in management positions. Regarding job positions, all interviewees held leadership positions, either in the administration of healthcare entities (RHA, PHCG, HHC, and LHU) or as City Council mayors. Additionally, four interviewees were clinicians (e.g., physicians or nurses) within the entities they represented.

Data analysis was conducted based on the previously identified categories and subcategories, as presented in the results below. Overall, no differences were observed among the three case studies, except for regional variations noted in the subsections “Difficulties in providing care to older adults,” “Adaptation of care to the WHO Principles,” “Desirable changes in care provision,” and “Main limitations to implementation.”

### 3.1. Familiarity

#### Acquaintance of WHO Principles

Out of the total protocols, it is noteworthy that only four interviewees mentioned awareness of the age-friendly healthcare concept, and even fewer, only two, specifically mentioned familiarity with the WHO Principles.

While the interviewees could not precisely outline the content of the WHO Principles, they did grasp the overarching theme, which encompasses “the entire issue of user safety, including polypharmacy management, comorbidity management, and active aging management”. They aim to ensure “the preservation of individual autonomy and independence for as long as possible, prioritizing quality of life and extending the years of healthy living”, in the words of one of the interviewees. Additionally, they emphasized the significance of delivering healthcare services to older adults within their homes and involving families in the caregiving process. Despite most interviewees being initially unfamiliar with the concept, they expressed that, once explained, the principles are consistently considered by healthcare providers in their daily interactions with older adults.

Furthermore, four interviewees claimed familiarity with the guidelines outlined in the NSAHA, especially concerning the application of the WHO Principles in PHC and their dissemination to HHC. In this context, one respondent commented that “the document is quite interesting; it delineates the actions and entities that should participate in the implementation and support its realization”.

Regarding their awareness of concepts derived from the WHO Principles (e.g., age-friendly hospitals, age-friendly healthcare system, and age-friendly emergency service), all respondents primarily focused on the concept of age-friendly cities, emphasizing the need for collaborative efforts between local authorities and healthcare services. Additionally, two interviewees believed that the Age-Friendly Principles were synonymous with those of the Age-Friendly Cities project.

Table 2 summarizes the obtained results regarding the interviewees’ acquaintance with the subject under investigation.

### 3.2. Current State

#### 3.2.1. Difficulties in Providing Care to Older Adults

The primary challenge highlighted by the interviewees in providing healthcare to the older population pertains to the insufficient number of human resources (Table 3). First, there is a shortage of healthcare providers available to manage the proposed activities and projects, including physicians, nurses, nutritionists, and psychologists. Second, this scarcity results in some older adults not having a family general practitioner or nurse. This absence hinders “continuity of care and comprehensive health monitoring for the elderly, as they are never seen by the same physician”, as expressed by one interviewee. In the case study conducted in the interior region, it was observed that older adults must undertake travel over long distances to access medical appointments, often lacking access to public transportation for such journeys. Similarly, in coastal regions, the closure of healthcare centers exacerbates the distances to be covered, consequently affecting healthcare accessibility. This shortage of human resources leads to a decline in the quality of care provided and an increase in waiting times for appointments. As one interviewee stated:

*“More and more, there’s difficulty in maintaining full schedules and having individuals who are part of the hospital staff. This leads to the need to rely on service providers who are less committed to the hospital and often lack the same level of expertise as those who would be dedicated staff. As a result, waiting times are exceedingly long overall, and there’s a significant tendency for the quality of medical care to diminish.”*

The second issue addressed by the interviewees is related to the healthcare management system (Table 3). A difficulty in this area concerns medication management. On one hand, there is the challenge of battling polypharmacy, which is driven by various specialist medical appointments that do not view older adults as a whole, and, on the other hand, there is low therapeutic adherence due to the difficulties in taking medications or financial incapacity to purchase them. Similarly, a significant number of hospitalizations occur without clinical justification due to a lack of a familial support network. This underscores the absence of an integrated response from social and healthcare services. Additionally, there is some difficulty in preparing caregivers of older adults for postdischarge care at home. The interviewees also mentioned the lengthy waiting lists for initial medical appointments and surgeries as one of the issues in providing healthcare to older adults.

The third problem highlighted by the interviewees was related to the lack of preparedness in the infrastructures of care providers for the older population (Table 3). These facilities lack proper physical accessibility, heating, or hot water. Moreover, there’s an insufficient number of hospital beds and limited space for service expansion. In this context, the following example was given by one of the interviewees, involving two healthcare centers on the coast of Portugal:

*“One of them is spread across the ground floor and the first floor without an elevator, presenting an immediate accessibility challenge for the elderly, as there are only stairs available. Another is a three-story building in the city center, also lacking an elevator, and the treatment room is situated on the first floor.”*

These accessibility issues primarily arise in coastal regions, as their facilities are situated in repurposed residential buildings adapted for healthcare provision. Since structural challenges are hard to overcome, “the Regional Health Administration aims to close all these units and open new ones”, as stated by an interviewee.

Another issue addressed pertained to professional training (Table 3), where a lack of humanization in healthcare was reported. This implies that clinicians, technical, and administrative staff are not adequately prepared to interact with older adults, failing to respect their decision making and support their involvement in care.

It is also worth noting the significant impact that the pandemic had in this context (Table 3), as it compromised the follow up of chronic patients, home visits, preventive screenings, and surgical procedures. In this regard, the interviewees indicated that the greatest impact of COVID-19 was felt in the interior regions of Portugal, exemplified by:

*“We still have a highly unequal country, with very pronounced disparities between the interior and more developed areas. Therefore, I am fully aware that the opportunities for those living on the coast were completely different from those living in the interior. I remember hearing a colleague talk about the difficulties even in the vaccination process, as the elderly had to respond to a message and didn’t even have network coverage.”*

#### 3.2.2. Changes in Providing Care to Older Adults

According to the interviewees, the main change in healthcare (Table 4) for older adults in the last decade concerns the introduction of digital technologies in the healthcare field. On one hand, this represents the advancement of science in the development of diagnostic and therapeutic equipment, innovative medication production, chronic disease management, and rehabilitation. On the other hand, it reflected the COVID-19 pandemic, in which there was a significant increase in the use of online resources and telephone communication, whether for medical appointments or monitoring of hospitalized family members. In this regard, one interviewee mentions that technologies are advantageous for carrying out surveillance medical appointments but should not restrict access to healthcare for acute cases, as personal contact remains an excellent tool for assessing nonverbal cues.

The possibility of homecare was the second most discussed aspect by the interviewees, particularly home hospitalization. For instance, “the hospital has incorporated home hospitalization into routine activities, increasingly reaching a consensus that the home is where healthcare providers should operate in an organized and continuous manner”, according to one interviewee. Furthermore, the potential for providing palliative care at home and increasing home visits for treatment or monitoring was emphasized.

The interviewees also highlighted the construction of new healthcare facilities in the last decade and the improvement of accessibility to existing ones. They also mentioned the creation of the network for continuous care, although with a substantial waiting list for medium-term care units, and the establishment of a palliative care network as an advantage in age-friendly healthcare. Additionally, improvements in hospital referral networks were noted.

In a complementary manner, the introduction of generics in Portugal significantly lowered medication prices, which means that “the pharmacology weapon has become generalized and accessible to everyone, because whether we like it or not, we have an elderly population with limited resources, and the main expense is medications”, according to one interviewee. The provision of dental vouchers for older adults, i.e., free access to oral healthcare for low-income NHS users, facilitated easier access to oral health appointments and treatments. The increase in older adults’ literacy also led to higher expectations regarding the quality of healthcare provision and the clinician–patient relationship. Furthermore, in some healthcare centers, movement rooms were established to facilitate supervised and guided physical exercise for older adults, along with health-literacy sessions. 

In contrast, there was an increase in the duration and number of hospitalizations among older adults due to multiple pathologies and situations of polypharmacy.

### 3.3. Initiatives

#### 3.3.1. Age-Friendly Policy

The entirety of the interviewees acknowledged that an age-friendly healthcare policy is not implemented (Table 5). In this regard, one of the interviewees mentioned that despite being an interesting project, in practice, it cannot be implemented in the NHS because there is no planning, no visible outcomes, and no assessment.

However, all interviewees identified the need to prioritize the adaptation of healthcare for older adults (Table 5), given that “comorbidities and the worsening of the clinical condition increase the demand for healthcare services by older adults”, according to one interviewee. Similar to baby-friendly healthcare centers accredited by the United Nations International Children’s Emergency Fund (UNICEF), the importance of implementing a differentiated policy for older adults that “allows optimization, efficiency, and effectiveness of NHS resources” was emphasized by an interviewee. To achieve this, one of the interviewees considered it necessary to involve all sectors of society because “having good environments, good professionals, and good organization in the healthcare sector alone is not enough.” Similarly, an interviewee stated that existing projects should be continued and a deep reform in the NHS should be encouraged to create new projects that address the needs of older adults. Nevertheless, one interviewee mentioned that “before directing this policy or healthcare policy reform towards older adults, I think it has to be for the general citizen, for any citizen of any age, and that then reflects on the provision of care to older citizen.” 

Still, in this context, interviewees from the interior region mentioned that adopting an age-friendly policy would not only be beneficial for residents but also for attracting tourists to their territory and promoting economic development, as exemplified by the following statement:

*“For anyone coming from the United States of América or England to spend a week here, the tourist operators certainly consider the healthcare conditions in place, especially cardiology, because older adults usually fear that something might happen.”*

#### 3.3.2. Projects for Age-Friendly Healthcare

In this subtheme, it was verified that the majority of the interviewees indicated the presence of implemented projects for the older population in healthcare or the community in collaboration with them (*n =* 9). The remaining interviewees mentioned that they do not have specific projects for the older population, but they pay special attention to this age group in their daily activities (*n* = 2). The three case studies presented initiatives focused on older adults within the scope of healthcare. Considering the WHO Principles, it is possible to state that the majority of the projects concern the healthcare management system principle, followed by the information, education, communication, and training principle, as well as the physical environment principle, in equal measure (Table 6). Additionally, there are projects in the rehabilitation and physical activity area, as well as in physical safety, that do not align with the WHO Principles.

In the principle of the healthcare management system, the respondents highlighted home-care services provided by a multidisciplinary team (physician, nurse, psychologist, physiotherapist, and social worker) from healthcare centers to the homes of older adults, where healthcare and caregiver support are rendered. Within this context, one of the coastal case studies is currently implementing a pilot project aimed at integrating home-care services from the healthcare sector with the social sector, seeking to provide personalized and tailored responses to older adults through a collaborative effort involving PHC, PHCG, HHC, RHA, social security, and local authorities. Additionally, within the home setting, a project focusing on palliative care within a family environment is notable in the interior region.

In the domain of PHC, surveillance medical appointments for chronic diseases are conducted every three months. A telephone hotline has been established to record contacts and provide same-day callbacks. The Barthel scale is applied to all older adults to assess the need for homecare. HHC settings have developed specific performance indicators (e.g., reduction in anxiolytic usage and prescription medication in older patients), standardized procedures for treating ulcers and wounds, and a nutrition medical appointment for older inpatients. Furthermore, in the interior region, within a hospital context, a project in collaboration with pharmacies has been implemented. When an older adult visits the emergency department between 10 PM and 8 AM and has a prescription, the pharmacy delivers the medication to the hospital.

Lastly, although not directly centered on the older population, an interviewee mentioned the implementation of a ‘Local Health System’ along the coast. This system envisions the integration of healthcare centers, hospitals, and public and private institutions with direct or indirect involvement in healthcare to promote health, ensure continuous care provision, and optimize resources. This system holds the potential for age-friendly healthcare.

Regarding information, education, communication, and training, caregiver training projects during home visits stand out. These cover topics like “mobility of bedridden older adults, nutrition tailored to older adults with swallowing difficulties, management of chronic medication”, according to one interviewee. Similarly, older adults receive training in their homes on “fall prevention, medication management, physical exercise, reducing salt intake, diabetes prevention, and stroke prevention”, according to another interviewee. In some cases, healthcare providers from community-care units, local administrative bodies, senior universities, or social institutions provide this training. Continuous training is also provided to healthcare providers on prevalent chronic conditions in older adults (diabetes and hypertension), as well as training for administrative staff in age-friendly services.

Regarding the physical environment, interviewees mentioned initiatives for improving accessibility in and around healthcare facilities, such as “sidewalk lowering, ramp installation, signage modification, elevator installation, replacement with automatic doors, and fully adapted restrooms”, according to one interviewee. Additionally, the interior case study is expanding emergency and inpatient services to enhance capacity and improve patient flow. New operation rooms with innovative equipment are being established. In terms of transportation, coastal case studies have bus lines stopping at HHC and PHC, while the interior case study offers on-demand transportation. Moreover, in the interior region, transportation is provided for older adults who need to travel between hospitals for diagnostic tests.

Finally, although not aligned with the WHO Principles, interviewees noted that municipalities, in partnership with PHC, are promoting physical activity programs. “Movement rooms” have been created within the PHC to facilitate physical activity, according to one interviewee. Furthermore, an innovative project is being implemented in one coastal case study involving “social prescription”, according to another interviewee. In this approach, during consultations, the physician identifies older adults’ needs and, based on prior information collection about local social resources, recommends institutions or local associations best suited to meet older adults’ needs. Another project involves providing an “SOS bracelet” to isolated older adults. In emergencies, they can activate the red button, which triggers a call to a telephone center that directs appropriate assistance. 

### 3.4. Implementation

#### 3.4.1. Adaptation of Care to the WHO Principles

All interviewees were invited to assess, on a scale of one to seven points (from terrible to excellent, respectively) the adequacy of healthcare units to WHO Principles. The care management system received the lowest evaluation [a median of four points (i.e., reasonable)], while the physical environment was rated with a median of five points (i.e., good). In the principle of information, education, communication, and training, the interviewees themselves differentiated their evaluations, assigning a median of five points to healthcare providers and three points (i.e., bad) to administrative personnel. Across the three case studies, in the principle of information, education, communication, and training, the south coastal case study achieved a median rating of six points (i.e., great), while the central coastal and north interior case studies obtained a median of five points. In the context of the healthcare management system principle, the interior region stood out with a median of five points, whereas the coastal regions had medians of three and four points. Concerning the principle of the physical environment, the north interior and south coastal regions both presented a median of five points, while the central coastal region exhibited a median of four points.

Overall, it was on the principle of the healthcare management system that the interviewees focused their statements, pointing out more positive aspects than negative ones, despite the initially lower evaluation of this principle. Considering the total number of mentions for each principle, it was at the level of the physical environment that the interviewees presented a more negative perception (Table 7).

In the principle of information, education, communication, and training, the interviewees mentioned that healthcare providers receive training on polypharmacy, management of comorbidities, violence prevention, and communication methods pertaining to older adults. Additionally, one of the interviewees stated that healthcare providers are entitled to fifteen days of training, with the choice of enhancing their skills in preferred areas, although administrative staff do not share the same privilege. Conversely, interviewees pointed out that the training sessions lack standardization, occur in insufficient numbers, do not encompass all providers, involve the same individuals attending repeatedly, and lack specificity regarding older adults. Furthermore, an interviewee indicated that moments of education and empowerment for older adults occur in healthcare units, albeit sporadically. On this note, an interviewee commented:

*“Healthcare providers in Portugal are highly prepared, I believe we have well-prepared healthcare providers (e.g., physicians and nurses). However, the conditions under which they work, the long hours, staff shortages, and the stress they experience contribute to care provision not being at their best in terms of their skills and abilities. They possess the skills, but they lack the circumstances, either due to overtime or continuous shifts.”*

Within the principle of the healthcare management system, the focus was on homecare, where therapeutic reconciliation, vaccine administration, support for informal caregivers, palliative care services, and 24 h home hospitalization were provided. Likewise, older adult patients were accompanied by a family member or caregiver during medical appointments, appointment reminders were issued the day prior, general information on abuse and violence was provided, weekly medication preparation was conducted in the ward, rooms were organized by age with two beds per bathroom, and a discharge note with a treatment guide for older adults and caregivers was issued. In this context, an interviewee emphasized:

*“With regards to older people, when they are hospitalized, we always try (unless they have a condition that prevents it) to explain the reasons, what we are going to do, the tests we request, and upon discharge, we explain the procedures to follow at home, the purpose of the medications, all these steps. When the individual lacks comprehension, we try to inform the caregiver or family member; we are careful about this in our services, as well as in outpatient consultations.”*

Another positive aspect highlighted regional differences between the interior and coastal areas. While the interior region noted a functional information-exchange system between primary and hospital care and internal referrals, the coastal regions faced difficulties in this process, especially hospitals accessing primary care information. Moreover, the interior region extended its healthcare centers’ hours of operation to accommodate emergencies due to the considerable distance to hospitals, in contrast to the coastal regions.

Concerning negative aspects, the absence of an individual care plan, lack of dedicated lines for older adults’ patients, geriatric emergency services, sufficient human resources in emergencies and healthcare centers, multidisciplinary teams, acute care units for older adults, access to clinical records of private healthcare facilities, and integration of healthcare services with social security were noted. Additionally, interviewees mentioned the absence of a geriatric specialty or case manager for older adults; in both cases, the role was assumed by internal medicine physicians in hospitals. However, in continuing care, a case manager position exists and is held by nurses.

In the principle of the physical environment, noteworthy aspects included replacing stairs with ramps, utilizing nonslip cleaning products, installing bathroom support bars, automatic doors at main entrances, elevators in multistory buildings, and expanding outpatient, inpatient, and emergency services. Conversely, issues included the lack of designated parking spots, inadequate diagnostic and therapeutic equipment in healthcare centers, the presence of asbestos roofs in some structures, and uphill locations of units exacerbated by weak transportation networks. On this matter, an interior-region interviewee affirmed that transportation to hospitals posed the greatest challenge, although local authorities offered organized transportation (i.e., scheduled and defined stops for collective travel) and on-demand services (i.e., individual, as needed). Coastal regions also highlighted transportation shortcomings, particularly in terms of schedule availability, which primarily aligned with school hours and did not meet the older population’s desired frequency. Another differentiation between coastal and interior regions is related to the age of buildings and corresponding accessibility conditions. In this regard, a coastal region interviewee stated:

*“We have residential buildings with old elevators, stairs, which obviously pose significant architectural barriers for older adults, unfortunately, we still have quite a few of them. As you know, all major cities have these issues, spaces are more expensive, and buildings are older (…) You’ll probably find spectacular healthcare centers in the country, well-built and age-friendly, but in major cities, you’ll find older healthcare centers in buildings that don’t have all the desirable conditions for age-friendly healthcare.”*

Another interviewee added about coastal units:

*“When a central hospital is housed in a 16th-century convent, no matter how many efforts are made, achieving accessibility, parking, and ease of access is impossible. Notice that most of our hospitals (or a significant portion) were likely built around the 1950s, when concepts were vastly different from today (…) For years, new hospitals haven’t been constructed, except recently when a new hospital was built with different accessibility. However, it’s still situated atop a hill where public transportation doesn’t extend to the hospital; only now, there’s discussion of installing a metro station within for easier access.”*

In contrast, an interviewee from an interior region stated:

*“Regarding physical infrastructure, we’re well served on two levels, let’s break it down. In terms of healthcare centers, I think in most municipalities, the physical structures of healthcare centers were renovated or newly built ten or fifteen years ago. For hospitals, there aren’t any poorly designed spaces (…) we have distinct realities; for instance, the department of continuing care, which includes palliative and convalescence units, is prepared differently; more specialized units, with multiple specialties, might encounter some issues in some services but improvement works are underway.”*

#### 3.4.2. Desirable Changes in Care Provision

In this subsection, the interviewees provided insights into the main changes to be implemented in healthcare, considering the shortcomings. In this context, the principle of the healthcare management system gathered the highest number of suggestions (*n* = 47), followed by the principle of the physical environment (*n* = 21), and the principle of information, education, communication, and training (*n* = 19) (Table 8). When excluding repeated ideas, the same hierarchy persists, with interviewees directing their interventions toward the principle of the healthcare management system. 

In the principle of information, education, communication, and training, all interviewees deemed it essential for healthcare providers (e.g., physicians of all specialties, nurses, psychologists, and nutritionists) to receive specific geriatric training, which could potentially be mandatory and integrated into the thirty-five hours of annual training they are entitled to. Noteworthy topics for training included the aging process and effective communication with older adults, although two interviewees suggested that nondiscriminatory physician–patient communication should be addressed at the university level. Alongside this, there was a consensus that training programs should be scientifically validated, showcasing potential areas of interest, and protocols should be established to monitor the practices that healthcare providers learn during training. Interviewees from coastal regions also touched upon training for technical assistants, “who are the first contact for older adults within the NHS”, necessitating an assessment of needs and tailored training sessions, focusing on techniques for assisting older adults with impaired hearing or comprehension. These interviewees also emphasized the development of educational sessions for older adults and caregivers in health-literacy areas (e.g., diabetes and hypertension prevention, physical exercise, and healthy eating), going beyond the simple distribution of information leaflets. Lastly, one interviewee stated the importance of “educating older adults about the range of healthcare services available at each level, to aid in selecting the appropriate service when needed and thus avoid unnecessary trips or overcrowding of services without justification.”

Within the principle of the healthcare management system, the necessity for well-planned projects under the framework of age-friendly healthcare was discussed—detailing factors like the number of healthcare providers required, resources, assessment, and monitoring—to ensure projects progress beyond the conceptual stage. Subsequently, the urgency of increasing human resources and reducing the patient load per physician emerged as a predominant concern. An interviewee highlighted:

*“As for hours, increasing them isn’t feasible because we already have a 40-h workweek (which is more than what most healthcare providers have). To resolve this, there’s only one solution, and that’s increasing the number of healthcare providers, thereby reducing the number of patients each is responsible for. If I have one thousand patients, I can manage tasks in forty hours that I can’t accomplish in forty hours with two thousand patients—it’s not humanly possible or practical.”*

Other aspects addressed encompassed the presence of a social animator in waiting rooms, availability of formal and informal (e.g., volunteers) healthcare providers to guide and assist older adults through the services, the creation of guidance circuits throughout services, offering a telephone helpline to prevent unnecessary travel, expanding telemedicine, acquiring modern medical equipment, decreasing the number of beds per ward, expanding the National Network of Integrated Continuing Care, prioritizing appointment scheduling, sending appointment reminders, extending opening hours to accommodate family accompaniment, increasing consultation time, and regularly conducting community screenings. Several proposals were suggested for homecare, such as increasing joint healthcare and social care visits, bolstering human resources for these visits, namely, to evaluate and suggest environmental changes, providing complementary diagnostic tools, and conducting therapeutic reviews by community pharmacists. In this context, two interviewees stood out:

*“This is still a cultural aspect: it’s not the physician who goes to the person, the physician only visits at home in emergencies. Generally, we chase after the healthcare provider rather than the other way around. This is a cultural hurdle that needs to be overcome because healthcare can be provided at home with the same quality as in a clinic.”*

*“Another aspect of integrated care, which is being discussed a lot now, involves the interaction between healthcare (primary and hospital care) and Social Security. If the aim is to keep an older adult patient at home, certainly much more than healthcare aspects will be required—nutrition, household cleaning, provision of a variety of necessities. These entities need to be in harmony and work together cohesively.”*

In the same domain of healthcare management, interviewees proposed appointing a coordinator or case manager for older adult patients, establishing a multimorbidity consultation to prevent appointments with different specialists on consecutive days, implementing a chronic prescription quality-improvement program by incorporating medication modules into patient clinical records to mitigate cases of polypharmacy, and enhancing clinical information systems for care coordination and avoiding redundant tests. Another frequently raised aspect was the creation of a geriatric emergency or fast track within the Manchester Triage System for older adult patients, similar to existing protocols for heart attack, stroke, and trauma, with qualified geriatric personnel. Finally, the interior-region case study emphasized the need for providing immediate life support ambulances or prehospital units in remote healthcare areas (where older adults reside) to ensure swift and urgent responses to the population, alongside incentives to attract physicians to rural regions, enabling the advancement of proposals aligned with age-friendly healthcare.

In the principle of the physical environment, interviewees suggested ergonomic benches in waiting rooms, ramps throughout interior spaces along with elevators and stairs, Z-shaped ramps at main entrances, bathrooms with support bars, light and spacious doors, wide and automatic doors in all services, free water dispensers, public telephones, vending kiosks, and recreational areas for older adults. Furthermore, the need for relocating healthcare centers situated within residential buildings was emphasized, as well as increasing the number of parking spots for individuals with reduced mobility, boosting the availability of vehicles for home visits, and allocating a budget category in PHCG budgets dedicated to physical space rehabilitation to facilitate adaptation works. On this matter, an interviewee mentioned, “I can’t undertake a bathroom renovation if it isn’t budgeted for, or I can, but I have to deduct it from the medication budget, which is budgeted for. But I can’t remove the medication because patients need it, so I can’t.” Lastly, the interior region underscored the need for creating an affordable, regularly scheduled healthcare transportation network, offering free services to low-income older adults. Additionally, the suggestion of organized transportation by local authorities to take older adults to HHC or PHC was put forward. Coastal regions suggested the creation of a door-to-door transportation service for older adults with limited mobility. 

### 3.5. Means 

#### 3.5.1. Favorable Conditions for Implementation

In this subtheme, the interviewees were asked to identify favorable conditions for implementing the WHO Principles within the NHS (Table 9). In this context, the interviewees primarily focused on aspects related to networking, both among healthcare providers and between them and community stakeholders, notably firefighters (for transportation) and local authorities (for access to care and space requalification). They subsequently addressed management-related aspects, including the presence of electronic health record systems for communication between primary and hospital care, the creation of an LHU for integrating primary and hospital care, holding meetings involving healthcare organizations, the Ministry of Health, Central Administration, and RHA to identify needs, and including intermunicipal communities in hospital board meetings for participatory decision making. The training of healthcare providers was also mentioned, with reference to the existence of training centers within the PHCG, training of clinicians in geriatrics for palliative and continued care, and facilitating professional internships within organizations to augment human resources dedicated to projects. Concerning the physical environment, emphasis was placed on establishing new healthcare centers with the support of community funds and program agreements with the Government and the Ministry of Health, along with the environmental fund for acquiring electric vehicles. In this context, the southern coastal region underscored differences in the allocation of community funding, noting that their region seemed less favored compared to the others. Lastly, the interviewees highlighted the added value of the WHO toolkit formulation and suggested the creation of a national program with recommendations to facilitate the implementation of the WHO Principles. 

#### 3.5.2. Main Limitations to Implementation

In addition to favorable conditions, the interviewees were asked to address the main limitations (Table 10). The greatest number of limitations were found at the level of human resources. On one hand, they mentioned not having the time to dedicate to new projects or specific training due to the high number of affected older adults. On the other hand, they criticized the low number of healthcare providers in the NHS (such as physicians, nurses, psychologists, nutritionists, and physiotherapists), attributing it to the high number of retirements and the lack of healthcare providers exclusively working in the public sector. Within this context, the interviewees complained that administrative boards lack autonomy to hire healthcare providers, as they often have to wait for authorization from the Ministry of Finance, which exacerbates the issue of human-resource shortages. Another aspect mentioned pertained to regional disparities. The interior region noted that interior areas are most affected by the lack of human resources, as these territories are less attractive and lack policies to incentivize healthcare providers to settle there.

The physical environment was considered a limitation due to the lack of available space for improvements (e.g., recreational areas and specific parking spaces), as well as the insufficient number of vehicles to provide close healthcare services to older adults. Financial limitations were also discussed, particularly the low budget allocated to the healthcare sector, and the lack of financial autonomy for the PHCG, which are entirely dependent on authorization from the RHA. Lastly, the interviewees highlighted the lack of literacy among older adults, which hampers their engagement with programs and projects directed at them, and the lack of integration between community initiatives and healthcare services.

#### 3.5.3. Actors Involved in the Implementation

Lastly, the interviewees were asked about the actors involved in the implementation of the WHO Principles, and they highlighted actors from five main spheres: healthcare, social, political, academic, and security (Table 11). Among the direct actors, and with the highest number of mentions, the healthcare sphere included the Ministry of Health, Directorate-General of Health, RHA, PHCG, PHC, and HHC. As indirect actors, the political sphere stood out, which encompassed municipalities (local councils) followed by the social sphere, which included social solidarity institutions, parish centers, neighborhood networks, and the Institute of Social Security. The academic sphere included universities and polytechnics, and the security sphere included the National Republican Guard or the Municipal Police. 

## 4. Discussion

To address the five research questions formulated earlier, we defined categories and subcategories (as presented in Table 1), with each category corresponding to a specific research question. The preceding section presented the findings in a point-by-point manner. In this section, we discuss the results obtained for each question and compare them with international studies in the field.

The first research question centered on local and regional decision makers’ perceptions of the WHO Principles. The results indicate that the majority of respondents do not recognize the concept of age-friendly care, the WHO Principles, or their inclusion in the NSAHA. This limited familiarity is consistent with the findings of Tavares et al. [23], in which participants mentioned considering these principles in their professional practice only after receiving an explanation of what the WHO Principles entail. Moreover, the concept that resonated most with the interviewees pertained to the age-friendly cities project, which, despite being relatively recent, has already been translated into Portuguese in collaboration with national healthcare entities. Notably, this project has received significant investment from municipalities with the aim of integration into the World Network of Age-Friendly Cities [23]. The interviewees’ reduced familiarity with the WHO Principles may be attributed to the paucity of scientific publications in this area, as well as the fact that the NSAHA is merely a proposed framework outlined in legislation and has not received official approval from the Ministry [12].

The second research question sought to comprehend the current state of healthcare concerning the WHO Principles. In this regard, the interviewees highlighted key developments in healthcare favoring older adults in recent decades. Notably, in the Portuguese context, these advancements include the establishment of a comprehensive care network facilitating the coordination of healthcare services with social support for dependent individuals [31] and the construction of new healthcare facilities through public–private partnerships [32].

Consistent with trends in other international settings, interviewees also emphasized the introduction of digital technologies that have streamlined diagnosis and therapy while reducing the need for in-person healthcare visits [33]. Additionally, they underscored the importance of homecare, enabling older adults to remain in their homes for as long as possible [34], the establishment of palliative care networks to enhance the quality of life during end-of-life care [35,36], and the adaptation of physical environments to accommodate individuals with reduced mobility [37]. On this note, the interviewees identified several challenges in the current provision of care to older adults, echoing issues prevalent in healthcare systems worldwide, as supported by scientific research. These challenges include an inadequate number of healthcare professionals to manage care [38], situations of polypharmacy stemming from multiple prescriptions by different physicians [39], a shortage of hospital beds due to extended hospitalizations worsened by the lack of social support [40], the closure of healthcare facilities due to cost-cutting measures [41], issues related to accessibility [42], and the need for comprehensive training of healthcare providers to address the specific needs of older adults [43].

The third research question delved into the strategies presently employed in healthcare for older adults. Interestingly, none of the interviewees reported implementing an age-friendly policy within their healthcare organizations, even though they acknowledged the necessity of adapting healthcare to the needs of older adults. This finding aligns with a Portuguese study [23] and international research [44,45], indicating that divergent responses may arise from a lack of comprehension of the term “age-friendly”. Alternatively, it may stem from the perspective that older adults do not require specialized services solely based on their age but rather on their clinical condition [45]. Nevertheless, interviewees did highlight certain initiatives within their domains of older adult care, with a particular focus on those related to the principle of healthcare management systems. This emphasis may be attributed to the global priority placed on enhancing healthcare coordination and integration [46].

Among the various initiatives in Portugal, noteworthy examples include the hospital pharmacy’s delivery of medications, quarterly monitoring appointments at primary healthcare centers (PHCs), home hospitalization programs, the establishment of performance indicators specific to healthcare units for older adults, and educational training on healthcare issues targeting older adults and their caregivers within healthcare units or the community, as well as projects aimed at renewing and expanding physical healthcare facilities.

The fourth research question aimed to clarify the potential means of implementing the WHO Principles. Before identifying the desired changes, respondents evaluated the current alignment of care with these principles, generally offering a positive assessment. Remarkably, they praised the physical environment and the information, education, communication, and training of healthcare providers, rating them favorably. Nevertheless, respondents raised concerns about the physical environment.

This perception aligns with findings from a Portuguese study [19], which indicated that over half of the assessed criteria were not being implemented in a hospital in the central region. The same study highlighted persistent regional disparities, as many coastal hospitals were constructed in the 1950s, not accounting for Portugal’s demographic transition and lacking facilities suitable for older adults [19]. The interviewees lauded the positive attributes, such as well-appointed waiting rooms, ample lighting, access ramps, private offices, and wide doors to accommodate wheelchairs. These findings corroborate the results from international studies [13,47].

Conversely, interviewees pointed out drawbacks, such as the absence of dedicated treatment rooms for older adults, specific parking facilities, diagnostic equipment, and public transportation, which align with the outcomes of a study by Ahmadi et al. [15] conducted in the healthcare center of a developing country. Regarding the principle of healthcare management systems, interviewees reported a higher degree of implementation for certain criteria, including the availability of homecare, appointment reminders, medication plans for older adults and caregivers, and family-member support during consultations. These results resonate with international contexts and are supported by studies emphasizing the added value of implementing such criteria to provide age-friendly care [14,48]. On the other hand, they highlighted deficiencies in areas such as the absence of individual care plans for older adults, geriatric emergencies, multidisciplinary teams, and case managers—areas where other studies have identified positive aspects [49,50], but which are lacking in the Portuguese context.

Within the scope of the principle of information, education, communication, and training, interviewees noted the existence of training for healthcare providers in areas such as polypharmacy, violence management, and comorbidity management. However, they expressed dissatisfaction with the extent of training, the exclusion of administrative professionals, and the absence of specificity regarding older adults. These findings parallel those of a study conducted in Ontario [51], which found that education was only offered to a small group of workers, with physicians, nurses, and staff lacking training in verbal and nonverbal communication skills with older adults, the four geriatric syndromes, and preventive counseling.

After evaluating the criteria implementation, interviewees highlighted the desirable changes required to make healthcare more age-friendly, once again focusing on the principle of healthcare management systems. Within this principle, they proposed several improvements, including (i) adequate planning of projects for older adults to facilitate implementation, evaluation, and replication in various contexts; (ii) the availability of volunteers and sociocultural animators in waiting rooms; (iii) extended duration of medical appointments for older adults; (iv) longer unit opening hours; and (v) creation of case managers and multidisciplinary teams.

These suggestions align with the criteria outlined in the three subscales defined by McCusker et al. [48,52] for evaluating whether an emergency service is age-friendly. While international contexts have established geriatric emergencies, interviewees in Portugal propose the creation of a green lane for geriatric emergencies within general emergencies. They also emphasize the need to reinforce human resources, especially in interior regions, and advocate for the exclusivity of their work within the NHS.

In the realm of the principle of information, education, communication, and training, interviewees recommend that all healthcare providers receive training in geriatrics, with training plans being scientifically validated and their results evaluated. They also highlight the importance of older adults having access to health-literacy programs. Similar findings were observed in a study conducted in Hong Kong [13], which emphasized that staff knowledge about natural aging and its characteristics is essential when interacting with older patients. Additionally, staff training in communication skills was found to increase patience during interactions with older adults [13]. An Iranian study [15] concluded that many employees perceive aging as an illness, emphasizing the need to counter this negative attitude. Furthermore, international studies argue that physicians, nurses, and staff should receive training in geriatric syndromes, such as memory loss, urinary incontinence, depression, and falls/immobility [51,53].

In the context of the principle of the physical environment, interviewees suggest various improvements, including ergonomic benches in waiting rooms, elevators in all spaces, ramps at entrances, wide doors that open in both directions, and water dispensers. These recommendations align with various suggestions described in the literature [13,54]. The interviewees also propose the availability of electric vehicles for home visits and on-demand transport for healthcare organizations. Importantly, these aspects benefit not only older adults but all healthcare users, as supported by the opinions of those interviewed, which are consistent with several studies [44,47,48].

The final research question aimed to uncover the resources necessary for the proper operationalization of the WHO Principles. Interviewees identified several facilitators for providing age-friendly healthcare, including (i) collaboration between healthcare services, social support, and local authorities; (i) the use of electronic health records for sharing clinical data across different levels of care; integration of training centers into the PHCG; (iii) community funds allocated for the establishment of new healthcare centers; and (iv) acquisition of electric vehicles.

However, they also highlighted significant financial, space, and human-resource constraints. These challenges resonate on an international scale, as the lack of staff and high workloads have been cited by healthcare providers, older adults, and their caregivers as critical areas requiring improvement to deliver age-friendly healthcare [38,44,55]. Financial constraints play a pivotal role globally, making it challenging to adapt services to the WHO project, especially concerning modifications to the physical environment [56]. In addition to the restrictions felt by political decision makers and administrators, the potential advantages and disadvantages for older adults of applying the WHO Principles in the NHS were highlighted. Interviewees corroborated that the adoption of discriminatory measures in healthcare can have the reverse effect, further isolating the older population, limiting intergenerational sharing, and generating a feeling of paternalism, in line with Cooley’s study [57]. On the other hand, they positively highlighted greater autonomy and participation of the older adult in the care process, continued care focused on the older adult’s needs, greater family involvement in the older adult’s health, and an environment that reduces situations of delirium and hospital disorientation. These are aspects confirmed by other international studies [58,59,60].

In this process, various stakeholders play vital roles, including healthcare services, local authorities, social services, universities, and security forces, as mentioned by the interviewees. This network of collaboration reflects the “Health in All Policies” principle of the Declaration of Helsinki, which emphasizes that all public policies and decisions made across sectors and governance levels can significantly impact population health, health equity, and the ability of healthcare systems to protect and respond to healthcare needs [61]. Interviewees acknowledged the healthcare sector’s primary role while recognizing the specific and synergistic impact of actions by other sectors in society on health conditions and equity. This policy approach has also been adopted at the local level in North American and European countries, as indicated by Guglielmin et al. [62].

### Limitations

There are certain limitations in this analysis that warrant attention. First, the adoption of a case study approach may not offer a comprehensive representation of the broader context encompassing all public healthcare organizations in Portugal. Second, restrictions imposed by the Ethics Committee barred the collection of information that could potentially reveal the identity of the healthcare organization or the respondent, limiting the possibilities for region-specific, legal regime-based, or structural model-based analyses. Third, there is the possibility of social desirability bias influencing responses, although efforts were made to mitigate this by conducting interviews in private settings. Lastly, this study did not incorporate the older population as participants, and as such their experiences and needs remain unexplored.

## 5. Conclusions

Demographic changes in recent decades have prompted a growing emphasis on healthcare-system-level interventions aimed at promoting healthy aging and providing age-friendly healthcare. This study sought to assess the readiness of Portuguese NHS healthcare organizations for implementing the WHO Principles on this matter.

Our findings indicate that healthcare providers are not well acquainted with the WHO Principles, yet they exhibit a positive attitude toward the necessity of accommodating and delivering appropriate care for older adults. There is a notable need to translate the WHO toolkit [9] into Portuguese to bridge this knowledge gap. While strategies have been put in place to enhance healthcare provision for older adults in recent years, we have identified gaps in healthcare-provider training, the healthcare management system, and the physical environment. Accordingly, we have identified interventions to improve care availability and delivery, along with opportunities to strengthen age-friendly healthcare, acknowledging potential limitations and the roles of various stakeholders in society.

Among our findings, we highlight the proposal to establish a dedicated pathway for older adults within general emergency services and to bolster integrated continued care. Moreover, we recommend several measures that can be extrapolated to other international contexts. These include developing a comprehensive training program for physicians, nurses, and staff covering verbal and nonverbal communication strategies and the four geriatric syndromes. Creating health-literacy programs and preventive counseling for older adults and informal caregivers is also suggested. Furthermore, the implementation of case managers and multidisciplinary teams to assess and treat older adults holistically, thereby reducing polypharmacy and prolonged hospitalizations, is recommended. Adapting physical spaces to accommodate all users is another crucial aspect. Strengthening homecare services can minimize difficulties in accessing healthcare providers and reduce hospital-related syndromes. Finally, there is a call for mandatory cooperation between healthcare entities and local partners, as well as the creation of evaluation and monitoring tools for the WHO project [8]. Additionally, it is suggested that the WHO establish an accreditation system for age-friendly healthcare, similar to other global initiatives (e.g., the Global Network for Age-Friendly Cities and Communities and the Baby-Friendly Hospital Initiative). Such accreditation can boost the interest and motivation of health administrators to join this network and promote the integration of age-friendly principles into healthcare systems worldwide.

Looking ahead, we underscore the importance of adopting an interprofessional approach in future research, including various healthcare providers and stakeholders in the healthcare sector. Research should also delve into the needs, expectations, and challenges faced by older adults when accessing healthcare. Furthermore, assessing the effectiveness and impact of implementing WHO Principles in healthcare is essential. Lastly, we propose the formulation of quality indicators that can be used to develop checklists and assessment tools, ultimately strengthening public policies in Portugal.

## Figures and Tables

**Table 1:** Grid of content analysis of the interviews.

Categories	Aim	Subcategories
Familiarity	To understand the awareness about the WHO Principles.	Acquaintance of the WHO Principles
Current State	To understand how healthcare services are currently adapted to older adults.	Difficulties in providing care to older adults
		Changes in providing care to older adults
Initiatives	To realize which initiatives are being applied in the provision of healthcare to older adults.	Age-friendly policy
		Projects for age-friendly healthcare
Implementation	To assess how WHO Principles can be implemented.	Adaptation of care to the WHO Principles
		Desirable changes in care provision
Means	To recognize which resources are necessary for the correct operationalization of the WHO Principles.	Favorable conditions for implementation
		Main limitations to implementation
		Actors involved in the implementation

**Table 2:** Acquaintance of the studied topic.

	Number of Interviewees	
	Yes	No
Age-friendly healthcare	4	7
WHO Principles	2	9
National Strategy for Active and Healthy Aging	4	7
Derived Concepts		
Age-Friendly Cities	11	0
Age-Friendly Hospitals	0	11
Age-Friendly Healthcare System	0	11
Age-Friendly Emergency Service	0	11

**Table 3:** Main difficulties in providing care.

	Number of Mentions
Human resources	9
Healthcare management system	8
Physical environment	6
Pandemic	6
Training of healthcare providers	2

**Table 4:** Key changes in healthcare delivery in recent years.

	Number of Mentions
Digital technologies	7
Homecare	5
Infrastructures	4
Continuing and palliative care network	3
Pharmaceutical products	1
Social supports	1
Health literacy	1
Promotion of physical activity	1
Utilization of healthcare services	1

**Table 5:** Implementation of the age-friendly policy.

	Number of Interviewees	
	Yes	No
Age-friendly policy	0	11
Needs for healthcare adaptation	11	0

**Table 6:** Projects developed under the WHO Principles.

	Number of Projects
Healthcare management system	15
Information, education, communication, and training	9
Physical environment	9
Others	
Rehabilitation and physical activity	4
Security	2

**Table 7:** Implementation of the WHO Principles.

	Number of Mentions	
	Positive	Negative
Information, education, communication, and training	6	7
Healthcare-management system	20	16
Physical environment	10	12

**Table 8:** Changes in the provision of healthcare in line with the WHO Principles.

	Number of Mentions
Information, education, communication, and training	19
Healthcare management system	47
Physical environment	21

**Table 9:** Favorable conditions for the implementation of the WHO Principles.

	Number of Mentions
Networking	5
Healthcare management system	4
Physical environment	3
Training of healthcare providers	3
Strategic guidelines	2

**Table 10:** Main limitations to the implementation of the WHO Principles.

	Number of Mentions
Human Resources	12
Physical environment	5
Financial	3
Literacy	1
Networking	1

**Table 11:** Actors involved in the implementation of the WHO Principles.

	Number of Mentions
Health	11
Policy	10
Social	8
Academic	5
Security	1

## Data Availability

The data used to support the findings of this study will be available from the corresponding author upon reasonable request.
